# Exploring the Neural Correlates of Metal Exposure in Motor Areas

**DOI:** 10.3390/brainsci15070679

**Published:** 2025-06-25

**Authors:** Daniele Corbo, Roberto Gasparotti, Stefano Renzetti

**Affiliations:** 1 Department of Medical and Surgical Specialties, Radiological Sciences and Public Health, University of Brescia, 25123 Brescia, Italy; roberto.gasparotti@unibs.it; 2 Neuroradiology Unit, ASST Spedali Civili of Brescia, 25123 Brescia, Italy; 3 Department of Medicine and Surgery, University of Parma, 43121 Parma, Italy; stefano.renzetti@unipr.it

**Keywords:** metal exposure, neuroimaging, motor, MRI, manganese

## Abstract

Background and objective: Environmental and occupational exposure to toxic metals poses a significant risk to neurological health, particularly affecting motor-related brain structures. Essential metals like manganese, copper, and iron become neurotoxic when homeostasis is disrupted, while non-essential metals such as lead, mercury, and cadmium are inherently toxic, even at low exposure levels. We aimed to investigate the state of the art on neuroimaging evidence of the effects of exposure to toxic metals on motor related brain structures and functions. Methods: PRISMA guidelines were followed. We included studies that reported neuroimaging studies exploring the link between metal exposure and neural changes in motor areas. Results: We identified 518 papers, but only 20 articles were included. Our findings indicate that manganese is the most extensively studied metal in relation to the motor system using neuroimaging, but studies have also investigated the effects of other metals, including lead, mercury, and copper. Across these studies, the brain regions most consistently affected by metal exposure include the globus pallidus, caudate nucleus, frontal cortex, and cerebellum. Some studies exhibit structural or functional reductions in these areas that correlate with increased levels of metal exposure, suggesting a dose-dependent neurotoxic effect. Conclusions: This review synthesizes current neuroimaging evidence on metal-induced neurotoxicity, emphasizing its impact on motor function and highlighting critical gaps to guide future research and public health strategies.

## 1. Introduction

There is increasing concern regarding the adverse health effects associated with environmental exposure to toxic metals. These metals are prevalent in ambient air pollution, such as emissions from vehicles, as well as in contaminated soil and various occupational settings. Ecological studies have suggested that airborne heavy metals may act as cofactors in the development of motor neuron diseases, such as amyotrophic lateral sclerosis, through various mechanisms, including interference with enzymatic and metabolic processes or neurotoxic effects resulting from their excessive accumulation in the nervous system [1]. Essential metals, including copper (Cu), iron (Fe), and manganese (Mn), play crucial roles in biological processes. However, when their concentrations deviate from homeostatic levels, they can exert neurotoxic effects, potentially disrupting normal neurological functions [2]. Conversely, non-essential metals, such as cadmium (Cd), mercury (Hg), and lead (Pb), lack any beneficial biological role and are inherently toxic. Even at low exposure levels, these metals can cause significant harm to the nervous system. Environmental toxicants can negatively impact physical growth, cognitive abilities, and mental well-being. An increasing number of studies indicate that exposure to toxic elements like Pb is linked to long-term cognitive impairments, behavioral issues, and motor function issues [3]. In particular, it has been observed that neurodevelopment is particularly influenced by exposure to metals, especially motor functions. The motor network is a distributed system of cortical and subcortical regions responsible for planning, initiating, and executing voluntary movements. Key components include the primary motor cortex, premotor cortex, supplementary motor area, basal ganglia (BG), cerebellum, and spinal cord. These regions interact through complex excitatory and inhibitory pathways to coordinate precise motor output. Functional connectivity within the motor network is modulated by both intrinsic factors, such as neuroplasticity, and extrinsic influences, including sensory feedback and environmental demands. Mn is a vital trace element that supports numerous enzymes involved in energy metabolism, antioxidant defense, and neurotransmitter synthesis; however, excessive environmental or occupational exposure can overwhelm homeostatic controls, leading to Mn accumulation—particularly within neural regions such as the BG and resulting in oxidative stress, mitochondrial dysfunction, and motor and cognitive impairments [4,5]. Mn is the fourth most commonly used heavy metal globally, and prolonged exposure can lead to manganism—a neurotoxic condition primarily affecting motor function—commonly seen in occupations such as mining, welding, and ferroalloy production [6]. Additionally, numerous epidemiological studies have linked environmental Mn exposure from sources like industrial emissions, contaminated drinking water, and Mn-based pesticides to cognitive and motor impairments in both children and adults [7,8,9]. Given that inhalation is the primary route of Mn exposure relevant to human health [10], ultrafine Mn particles can deposit efficiently in both the nasopharyngeal and alveolar regions of the respiratory tract due to their high diffusivity. While systemic absorption through the lungs and subsequent transport via the bloodstream is a well-established pathway, an alternative and potentially more direct route for Mn entry into the brain involves the olfactory system. This pathway bypasses the blood–brain barrier (BBB), allowing Mn particles deposited in the nasal cavity to be transported along the olfactory nerve directly into the olfactory bulb (OB) and subsequently into deeper regions of the central nervous system (CNS). Evidence from rodent and other animal models supports this mechanism [11], demonstrating that metal particles can traverse the olfactory epithelium and gain access to the brain, thereby circumventing the protective constraints of the BBB. Magnetic resonance imaging (MRI) encompasses several modalities to study the brain. Structural MRI provides high-resolution images of brain anatomy, while functional MRI (fMRI) provides an indirect measure of neural activity by detecting changes in the blood-oxygen-level-dependent signal. Diffusion Tensor Imaging (DTI) maps white matter (WM) tracts by tracking water diffusion, and Magnetic Resonance Spectroscopy (MRS) assesses brain metabolism by detecting chemical concentrations. Voxel-Based Morphometry (VBM) is used to detect regional differences in brain tissue volume, particularly gray matter, enabling group-level comparisons in neuroanatomical studies. MRI serves as a valuable biomarker for exposure due to its capacity to detect Mn accumulation in the brain. Owing to its paramagnetic properties, Mn acts as a T1 contrast agent, meaning it influences the decay of the MRI signal following radiofrequency excitation of water molecules. In T1-weighted (T1W) images, brain regions with Mn buildup appear with increased signal intensity compared to unaffected areas.

Additionally, MRS can quantify various brain metabolites at millimolar concentrations, including gamma-aminobutyric acid (GABA), the primary inhibitory neurotransmitter in the CNS. Given its high concentration in the BG, GABA has been investigated as a potential biomarker for motor dysfunction associated with Mn exposure. Elevated thalamic GABA levels observed in Mn-exposed workers [12] have been linked to Mn accumulation in the substantia nigra, a region with GABAergic projections to the thalamus and dopaminergic projections to the striatum. While Mn neurotoxicity is known to impair nigrostriatal dopaminergic pathways—particularly by reducing D2 receptor levels in the striatum without altering dopamine or D1 receptor levels—its effects on nigrothalamic circuits remain less clear. The reduction in D2 receptors may disinhibit the indirect pathway, leading to increased inhibition of the thalamus and elevated thalamic GABA levels [13]. This mechanism aligns with observed motor deficits and suggests that Mn-induced disruption of BG–thalamocortical communication may underlie early neurotoxic effects.

DTI assesses WM integrity by evaluating the extent to which water molecules can diffuse within tissue. When water diffusion is more directionally constrained along fiber tracts, fractional anisotropy (FA) values are higher, indicating greater fiber organization. Conversely, lower FA suggests reduced structural coherence. Studies have shown that welders exhibit decreased FA in regions such as the corpus callosum (CC), frontal WM, and BG, with these reductions being associated with impaired fine motor control, as measured by synergy indices [2].

To date, only one morphometric study [14] has reported significant findings, revealing reduced brain volume in the globus pallidus (GP) and cerebellar regions among welders.

Structural and functional alterations in the BG, cerebellum, and frontal cortex—regions essential for motor learning, coordination, and executive control—can translate into measurable behavioral impairments. Damage to the BG may disrupt motor planning and execution, leading to slowed movements or tremors. Cerebellar changes can impair balance, fine motor skills, and motor adaptation. Alterations in the frontal cortex may affect attention, decision-making, and inhibitory control. These regions form interconnected circuits, so dysfunction in one can cascade through the network, amplifying deficits.

The aim of this review is to synthesize and critically evaluate current neuroimaging evidence on the effects of environmental and occupational exposure to toxic metals—both essential and non-essential—on motor- related brain structures and functions. A particular focus is posed on how metal- induced neurotoxicity manifests in regions associated with motor functions, including the BG, cerebellum, and WM tracts. This review also highlights gaps in the literature and aims to inform future research directions and public health policies addressing metal neurotoxicity.

## 2. Materials and Methods

### 2.1. Study Experimental Design

This narrative review was conducted following a re-adaptation of the PRISMA flow [15]. The research was performed in the period between September 2024 and January 2025.

### 2.2. Inclusion Criteria

This narrative review aimed to include all research studies that met specific criteria; they had to be published in English and available in full-text format. There were no limitations regarding participant age, publication year, or study design. Studies involving animal subjects were excluded.

### 2.3. Study Selection

**Figure 1:**
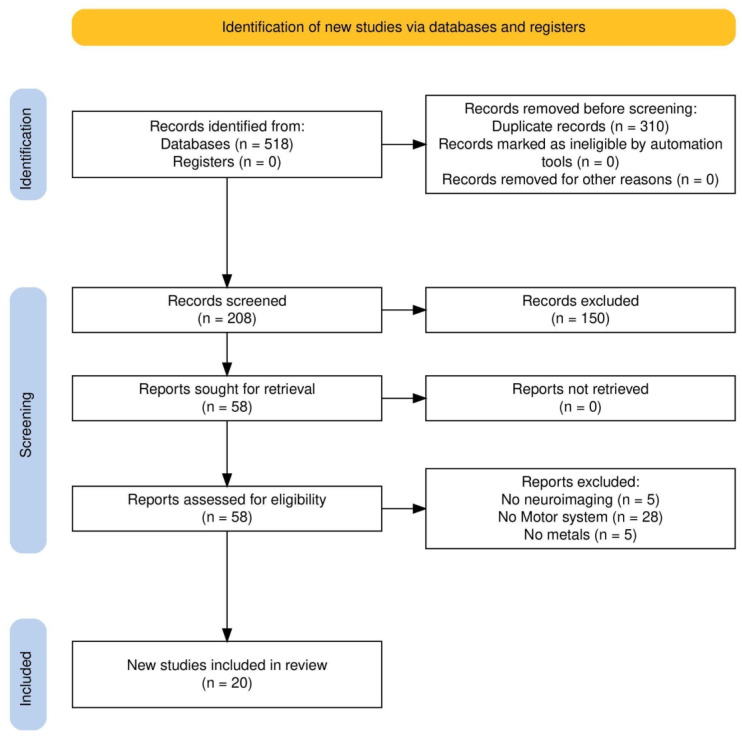
Flowchart of the narrative review process. Starting from 518 studies, 310 records were discarded because of duplicates, 150 were excluded because they partially covered the topic of interest, and 38 further studies were removed after the review of the full text because of missing information, leading to a total of 20 works included in the final narrative review.

To assess the relevant literature in this field, this qualitative review included works published up to January 2025, identified through a search of open-access databases such as PubMed, Google Scholar, Web of Science (WoS), and Scopus. The initial search in all databases utilized a combination of the following terms: [(environmental exposure OR metal exposure) AND (neuroimaging OR fMRI OR MRI) AND motor] without specifying a range time of publication dates. In addition to the initially identified articles, we examined for possible inclusion references from those studies, other relevant sources, and literature reviews. Two authors (DC, SR) conducted the entire selection process, which included choosing databases, applying exclusion criteria, performing secondary searches, and selecting final articles, as illustrated in the flow diagram (Figure 1). The initial search produced 518 results. After removing duplicates, 208 unique articles remained and were screened based on their titles and abstracts. Of these, 150 were excluded for not addressing all key topics, metal exposure, the motor system, and neuroimaging. The remaining 58 articles underwent full-text review, and their reference lists were checked for additional relevant studies, though no new articles were identified. A second full-text screening of these 58 articles was conducted to ensure compliance with the inclusion and exclusion criteria, leading to the removal of 38 articles (5 unrelated to neuroimaging, 28 unrelated to the motor system, and 5 unrelated to metal exposure).

From each included study, the following data were extracted: (1) authors and publication year; (2) sample size; (3) participant demographics; (4) metals; (5) neuroimaging methods and outcome measures; and (6) key findings.

## 3. Results

### 3.1. Manganese

The largest portion of the selected studies (N = 13, 65%) investigated Mn-related neural changes (Table 1).

**Figure 2:**
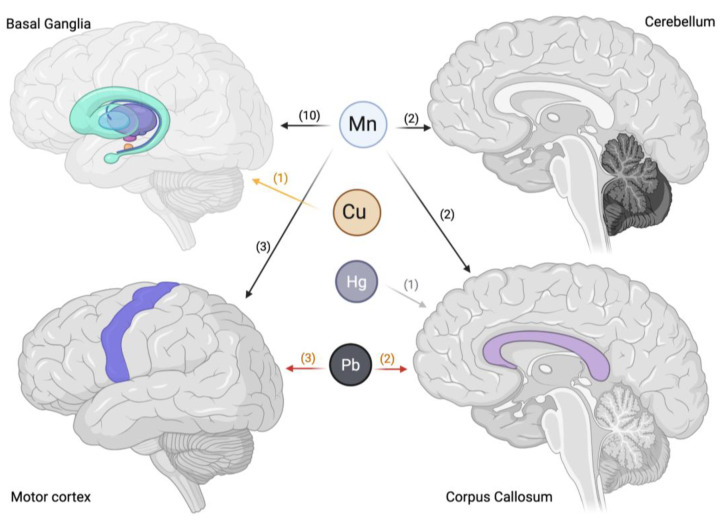
Principal neural correlates in motor area of metal exposure. The number of studies supporting the data is indicated in brackets (created in BioRender. Corbo, D. (2025) https://BioRender.com/7j7pdzw).

Among all metals, Mn stands out as the most extensively investigated for its neurotoxic properties, especially in relation to its accumulation in motor-related brain regions like the BG (Figure 2), which is implicated in disorders such as manganism and Parkinsonism. Due to the widespread public health concerns associated with Mn exposure, research has extended beyond occupational settings to include individuals exposed through environmental or other non-occupational sources. These studies encompass not only adults but also vulnerable populations such as adolescents and children, who may be particularly susceptible to its harmful impact on brain development and function. Among these studies, inhalation is the most commonly explored route of Mn exposure, highlighting that internal concentrations can be elevated not only by dietary intake but also by Mn emissions.

#### 3.1.1. Adolescents

In their study, De Water et al. [16] investigated the relationship between Mn exposure during critical developmental periods—specifically the prenatal, early postnatal, and childhood stages—as measured by dentine biomarkers, and the intrinsic functional connectivity (iFC) of the adolescent brain. By leveraging dentine as a temporally resolved biomarker of early-life Mn exposure, the analysis aimed to identify how variations in Mn levels across these sensitive windows may influence the organization and integration of large-scale brain networks during adolescence, a key period for neurodevelopment. The study found that higher Mn concentrations during the early postnatal period were associated with decreased iFC between key motor-related brain regions, including the right putamen and the precentral and postcentral gyri, in adolescents. Among various subcortical structures, the BG have been extensively investigated as potential anatomical biomarkers in MRI studies of Mn toxicity. Due to their central role in neurodevelopment, these structures are especially relevant when examining school-age children chronically exposed to Mn via drinking water. In this context, Lao et al. [17] applied a three-dimensional surface-based morphometry approach to assess key BG regions—the putamen, GP, and caudate—in children with prolonged Mn exposure. Their analysis revealed a statistically significant enlargement of the putamen, along with trends indicating increased volume in the left GP. Importantly, these morphological alterations were significantly correlated with poorer motor performance, suggesting that disruptions in the motor circuitry may underlie the observed neurofunctional deficits.

#### 3.1.2. Welders

Occupational exposure to Mn, particularly among welders, has been extensively studied for its neurotoxic effects, with a focus on both structural and functional brain alterations. Changes in WM integrity are among the most investigated aspects.

Kim et al. [18] employed DTI to assess whether welders chronically exposed to Mn exhibit microstructural differences in WM compared to non-exposed control subjects. Using an investigator-independent, voxel-wise analysis, the study revealed significant reductions in FA within the CC and frontal WM regions of Mn-exposed individuals. These FA reductions were accompanied by pronounced increases in radial diffusivity (RD), with minimal alterations in axial diffusivity (AD), suggesting that the observed microstructural changes are primarily attributable to demyelination rather than axonal degeneration. Although the GP is recognized as the primary site of Mn accumulation in the brain, the findings indicate that Mn-induced disruption of the CC may contribute to both clinical and subclinical motor impairments observed in chronically exposed welders. Furthermore, correlation analyses between DTI-derived metrics and neurobehavioral performance support the hypothesis that widespread WM abnormalities, particularly in the CC, are associated with subtle motor deficits. Mn shortens the T1 relaxation time in MRI, thereby increasing the signal intensity on T1W images and elevating the T1 relaxation rate (R1).

In a study by Sen et al. [19], region-specific Mn accumulation and its potential functional implications were investigated in individuals with subclinical levels of Mn exposure, such as welders. Volumetric analysis revealed no significant differences in the volumes of brain regions of interest (ROIs) between welders and control subjects. Moreover, no visually detectable hyperintensities were observed in the GP, putamen, or frontal gray matter (FGM) in any of the welders when assessed by conventional visual inspection. However, quantitative MRI analysis revealed significantly elevated R1 values in the OB of welders compared to controls. Additionally, welders exhibited significantly higher mean T1W signal intensities in the frontal WM, GP, and putamen. These findings suggest that Mn accumulates not only in the OB but also in multiple other brain regions, even in the absence of overt clinical symptoms. Importantly, the MRI-detected abnormalities were associated with subtle impairments in fine motor function, while cognitive performance remained unaffected.

Chang et al. [14] evaluated morphological brain changes in welders chronically exposed to Mn, with a particular focus on the relationship between structural abnormalities and subclinical motor dysfunction. Using VBM, they identified significant reductions in gray matter volume within the GP and cerebellum—regions known to be involved in motor control. Importantly, these volumetric reductions were found to correlate with subtle motor neurobehavioral impairments, suggesting that chronic Mn exposure may lead to region-specific neurodegeneration that precedes overt clinical symptoms.

Wu et al. [20] conducted another study that investigated the structural differences in workers exposed to Mn through VBM. The analysis revealed that, in comparison to HC, welders with Mn exposure exhibited significant alterations in GM volume, primarily localized in the medial prefrontal cortex, lentiform nucleus, hippocampus, and parahippocampus. The receiver operating characteristic (ROC) curve suggested that the hippocampus and parahippocampus demonstrated the strongest discriminatory potential in differentiating Mn-exposed individuals from healthy controls. Additionally, structural covariance analyses identified distinct connectivity patterns between the two groups, implicating key regions such as the thalamus, insula, amygdala, sensorimotor cortex, and middle temporal gyrus. Notably, the observed neuroanatomical changes showed no statistically significant correlations with clinical characteristics.

Long et al. [12] aimed to test the hypothesis that Mn exposure affects GABA levels in the brain and that such alterations may be associated with fine motor performance deficits in occupationally exposed workers. Using MRS, GABA concentrations were quantified and the relationship with both exposure duration and motor function was examined. Multiple linear regression analyses revealed a significant positive association between GABA levels and the duration of Mn exposure, suggesting a cumulative effect of Mn on inhibitory neurotransmission. Furthermore, GABA concentrations were inversely correlated with performance on all subtests of the Purdue Pegboard Test among smelter workers, indicating that elevated GABA levels may be linked to impaired fine motor skills. In addition to GABA alterations, the study also reported significantly reduced levels of myo-inositol (mI) in the thalamus and posterior cingulate cortex (PCC) of Mn-exposed workers compared to controls.

In the study by Ma et al. [13], welders with higher levels of Mn exposure showed elevated thalamic GABA concentrations and higher UPDRS3 scores compared to those with lower exposure and to control participants. While thalamic GABA levels were also associated with R1 values in the substantia nigra and frontal cortex, no direct correlation was found between GABA levels and UPDRS3 scores. Thalamic GABA appears to vary in response to changes in workplace Mn exposure, whereas UPDRS3 scores remain relatively unchanged.

The study by Lotz et al. [21] investigated the impact of Mn exposure on fine motor function in occupationally exposed individuals. To assess Mn accumulation in the brain, all participants underwent T1W MRI, with the R1 used as a quantitative biomarker of Mn deposition, particularly in the GP and substantia nigra—regions known to be susceptible to Mn accumulation. Despite the use of these sensitive imaging markers, the study found no significant association between R1 values in these regions and performance on fine motor function tests.

Monsivais et al. [22] employed high-resolution three-dimensional MRI and R1 relaxation mapping to systematically investigate regional variations in Mn deposition. Utilizing voxel-based quantification and statistical parametric mapping, the study delineated Mn accumulation patterns and examined their associations with neuropsychological and motor performance metrics. Occupational Mn exposure results in extensive accumulation beyond the BG, affecting cortical regions involved in motor functions. Mn deposition exhibits distinct regional associations, with exposure-related accumulation prominently observed in the cerebellum and frontal cortex, whereas motor function impairments correlate with Mn burden in the cerebellum and hippocampus.

Thunberg et al. [23] conducted a whole-brain mapping study to systematically investigate the impact of various welding processes on Mn accumulation in the brain. To assess potential differences in Mn deposition patterns, welders were stratified into three primary groups based on their welding method, and the R1 was measured utilizing quantitative MRI. The findings revealed that welders employing shielded metal arc welding exhibited lower total Mn accumulation in specific brain regions, including clusters within WM, the thalamus, putamen, pallidum, and substantia nigra, when compared with those using inert gas tungsten arc welding or continuous consumable electrode arc welding. Additionally, analysis of Mn levels in red blood cells (Mn-RBC) demonstrated a significant positive correlation with R1 values in a region encompassing the precentral and postcentral gyri, suggesting a link between circulating Mn concentrations and cortical Mn deposition. Furthermore, Mn-RBC levels were positively correlated with Mn deposition in the left primary somatosensory and motor cortices, reinforcing the notion that Mn exposure through occupational welding may have distinct neuroanatomical consequences depending on the specific welding technique utilized.

#### 3.1.3. Single Case

The neurotoxic effects of Mn exposure on motor-related brain regions can also be observed in individual cases of excessive occupational exposure. Alikunju et al. [24] reported a case involving a 40-year-old male with no preexisting comorbidities who had been employed as a welder in a steel factory for seven years without adequate personal protective equipment. The patient, with an upper- motor- neuron- type weakness on the left side of his face and spastic dysarthria, underwent an initial computed tomography scan, which revealed ill-defined hypodensities in the BG, though no evidence of hemorrhagic events was detected. Consequently, he was admitted to the stroke unit for conservative management and further diagnostic evaluations. Subsequent MRI of the brain demonstrated characteristic features consistent with Mn deposition and absorption, particularly within the GP and corticospinal tracts. These findings strongly supported a diagnosis of Mn-induced cerebral toxicity. Interestingly, despite the radiological evidence of Mn accumulation in the brain, the patient’s serum Mn levels measured at the time of admission remained within the normal range, highlighting the potential disconnect between circulating Mn concentrations and its neuroanatomical deposition.

**Table 1:** List of the selected studies in the narrative review that explored the association of Mn and the neural motor areas. Abbreviations: GABA, gamma-aminobutyric acid; GP, globus pallidus; Mn, manganese; MRI, magnetic resonance imaging; OB, olfactory bulb; BG, basal ganglia; R1, T1 relaxation rate.

Authors	Year	Functional Outcomes	Sample Size	Entry Route
Sen et al. [19]	2011	OB and other brain regions	14	Inhalation
Kim et al. [18]	2011	Brain microstructural abnormalities	49	Inhalation
Chang et al. [14]	2013	Changes in the GP and cerebellum	66	Inhalation
Long et al. [12]	2014	Alterations in GABA	32	Inhalation
Lao et al. [17]	2017	Altered BG neurodevelopment	23	Water
Ma et al. [13]	2018	Thalamic GABA	39	Inhalation
Lee et al. [25]	2018	Brain and functional changes	Review	Inhalation
de Water et al. [16]	2019	iFC	14	All
Lotz et al. [21]	2021	Fine motor functions and relaxation rates R1 in GP and substantia nigra	78	Inhalation
Wu et al. [20]	2022	Grey matter volume and structural covariance patterns	75	Inhalation
Alikunju et al. [24]	2023	Upper motor neuron type weakness on patient’s face left side and spastic dysarthria	1	Inhalation
Thunberg et al. [23]	2024	R1 measured using whole-brain quantitative MRI in an encompassing pre- and post-central gyri region	51	Inhalation
Monsivais et al. [22]	2024	High-resolution 3D MRI and R1 relaxation maps to identify Mn accumulation	59	Inhalation

#### 3.1.4. Review

In their comprehensive review, Lee et al. [25] examined the neuroimaging and behavioral consequences of low-level Mn exposure in welders. Their findings suggest that Mn accumulation in the brain may follow a non-linear trajectory, with significant increases in MRI-derived R1 observed only after a critical threshold of exposure is surpassed. This pattern implies a saturation-like effect, where Mn deposition remains minimal until cumulative exposure reaches a tipping point. Importantly, the authors highlight that R1 may serve as a more sensitive and dynamic biomarker for detecting short-term fluctuations in Mn accumulation compared to the traditional pallidal index (PI)—defined as the T1W signal intensity ratio between the GP and frontal WM. While PI has been widely used to assess Mn burden, its sensitivity to early or subtle changes appears limited relative to R1-based measures. In addition to functional imaging markers, the authors also discuss structural brain alterations associated with chronic Mn exposure. Specifically, DTI studies have reported reduced FA values in the BG, particularly in individuals with over 30 years of welding experience, suggesting long-term microstructural degradation in Mn-sensitive regions. From a behavioral perspective, the review emphasizes that traditional fine motor assessments may lack the sensitivity to detect early Mn-related motor impairments. Instead, more nuanced kinematic metrics—such as synergy indices that quantify movement stability and coordination—have been more effective in detecting subtle motor dysfunctions in exposed workers.

### 3.2. Lead

Some studies (N = 5, 25%) have investigated the relationship between Pb exposure and neural changes in motor areas (Table 2). These studies did not propose a clear hypothesis regarding the route of entry of this metal into the body, and the primary biomarker considered was blood, which reflects exposure from multiple sources.

Pb has been studied less extensively than Mn, although several studies have investigated brain alterations due to Pb exposure (Figure 2). Bleecker et al. [26] investigated whether chronic Pb exposure is associated with cerebral WMCs and whether these structural alterations mediate the relationship between Pb exposure and psychomotor slowing, as assessed by performance on the Grooved Pegboard (GrP) test. WM changes were identified as hyperintensities on T2-weighted MRI scans and were graded according to standardized criteria. To examine the association between Pb exposure and WMCs, logistic regression models were employed, adjusting for age and cerebrovascular risk factors. Pb exposure was quantified using several metrics, including integrated blood Pb (IBL), time-weighted average (TWA), and bone Pb (PbBn). The analysis revealed that WMCs were present in 23% of the MRI scans. After controlling for covariates, logistic regression showed significantly elevated odds ratios for WMCs in relation to all three Pb exposure metrics, indicating a robust association between cumulative Pb burden and WM pathology. Multiple linear regression analyses were then used to model both the direct effects of Pb exposure on GrP performance and the indirect effects mediated through WMCs. Among the exposure metrics, IBL exhibited the strongest direct association with GrP performance, suggesting that cumulative blood Pb burden may be particularly detrimental to psychomotor function. Furthermore, after adjusting for Pb exposure and covariates, WMCs provided additional explanatory power in detecting the effect on GrP performance, particularly in relation to PbBn. Path analysis confirmed that WMCs partially mediated the relationship between both PbBn and IBL with psychomotor slowing.

In their longitudinal neuroimaging study, Cecil et al. [27] investigated the long-term effects of childhood Pb exposure on adult brain structure using MRI. The study also examined how these structural alterations relate to historical neuropsychological performance. Whole-brain volumetric analyses revealed significant reductions in gray matter volume associated with higher mean childhood blood Pb concentrations. Applying stringent statistical thresholds, the researchers found that approximately 1.2% of total gray matter volume was significantly and inversely correlated with childhood Pb levels. The most pronounced volume reductions were observed in the FGM, particularly within the anterior cingulate cortex (ACC), a region implicated in executive function and emotional regulation. Notably, the extent and significance of Pb-associated gray matter loss were substantially greater in males than in females, suggesting potential sex-specific vulnerability to neurotoxic effects. Furthermore, fine motor function scores, derived from neuropsychological assessments, were positively correlated with gray matter volume in the cerebellar hemispheres. However, when the childhood blood Pb concentration was included as a covariate in the model, this correlation was attenuated, indicating that Pb exposure may partially mediate the relationship between cerebellar structure and motor performance.

Brubaker et al. [28] investigated the long-term effects of childhood Pb exposure on WM microstructure in young adulthood, using DTI to assess alterations in axonal integrity and myelin organization. The authors hypothesized that early-life Pb exposure would result in persistent disruptions to WM architecture, manifesting as region-specific diffusion abnormalities. Their analysis revealed significant Pb-associated reduced FA and AD, along with increased MD and RD, particularly within the corona radiata—suggesting compromised axonal coherence and demyelination in these projection fiber tracts. In the CC, including the genu, body, and splenium, the pattern was more complex: RD showed robust decreases associated with Pb exposure, while MD exhibited smaller, less significant reductions, and AD showed localized increases. These findings indicate that childhood Pb exposure may produce multiple, regionally distinct patterns of WM disruption in adulthood. The observed diffusion profiles suggest that both axonal and myelin-related processes are affected, with the nature and extent of the damage varying across different WM tracts.

Although the primary focus of the study by Seo et al. [29] was on working memory networks in individuals exposed to Pb, assessed using an N-back task during fMRI, the findings also have implications for motor system involvement. During the N-back task, participants were required to make motor responses, thereby engaging motor-related brain regions in addition to cognitive control areas. The study found that individuals with a history of Pb exposure exhibited significantly poorer performance on high-load working memory tasks compared to healthy controls. Functional imaging analyses revealed that, under increased cognitive demand, Pb-exposed subjects showed reduced activation in several key regions associated with executive function and motor planning. These included the dorsolateral prefrontal cortex, ventrolateral prefrontal cortex, pre-supplementary motor area, and inferior parietal cortex.

Takeuchi et al. [30] explored the relationship between body Pb burden and both gray matter microstructure and brain functional activity during cognitively demanding tasks. Drawing on a large cohort of typically developing young adults, the study integrated DTI, fMRI, and cognitive assessments to examine the subtle neurobiological effects of low-level environmental Pb exposure, as indexed by hair Pb concentrations. The findings revealed several weak but statistically significant associations. Higher hair Pb levels were linked to increased task-related activation in the right premotor cortex and pre-supplementary motor area during working memory challenges, suggesting a potential compensatory recruitment of motor-related regions under cognitive load. DTI analyses showed reduced FA in WM tracts adjacent to the internal capsule, indicative of compromised microstructural integrity. MD was decreased in dopaminergic projection areas within the left hemisphere and other distributed regions, possibly reflecting altered cellular density or neurochemical environment. Increased MD was observed in WM near the right fusiform gyrus, which may signal localized microstructural disorganization.

### 3.3. Mercury and Copper

Only two studies (10%) found relationships between exposure to other metals (Hg and Cu) and effects on motor areas (Table 3). These two studies considered inhalation and food intake as routes of entry, with the latter being a common source of exposure for Hg, particularly through fish consumption.

Compared to Mn and Pb, other metals have been less extensively studied using neuroimaging techniques, particularly with respect to their effects on motor-related brain regions (Figure 2). However, a notable exception is the study by Pujol et al. [31], which examined the neurotoxic impact of airborne Cu exposure in school-aged children. This study investigated environmental Cu exposure in educational settings and its association with motor function and brain structure. Higher levels of airborne Cu were significantly associated with poorer motor performance, suggesting a detrimental effect on motor system development. Structural MRI analyses revealed alterations in the BG, with the caudate nucleus showing reduced structural integrity. Specifically, the tissue composition and water diffusion properties—assessed via diffusion-weighted imaging—indicated a less coherent microstructural organization in this region. fMRI further demonstrated a consistent reduction in reciprocal connectivity between the caudate nucleus and the frontal cortex, implicating disrupted frontostriatal communication.

Migneron-Foisy et al. [32] investigated whether exposure to environmental contaminants is associated with microstructural alterations to the CC, a major WM tract involved in a wide range of cognitive, motor, and sensory functions. To characterize the CC’s microstructure, the authors employed diffusion-weighted imaging (DWI) and a tractography-based segmentation approach to analyze seven anatomically and functionally distinct ROIs within the CC. Multiple linear regression models were used to assess associations between DWI-derived metrics and concentrations of Hg and Pb, measured both prenatally (via cord blood) and postnatally. These models were adjusted for potential confounders, including sex, age, current alcohol and drug use, and intake of nutritional factors such as omega-3 fatty acids and selenium from fish consumption. The results revealed that both prenatal and postnatal exposure to Hg were significantly associated with increased FA in several posterior regions of the CC, including the anterior midbody, posterior midbody, isthmus, and splenium, where the most pronounced effects were observed. These increases in FA were primarily driven by reductions in RD, suggesting alterations in myelination or fiber packing density.

**Table 2:** List of the selected studies in the narrative review that explored the association of Pb and the neural motor areas. Abbreviations: CC, corpus callosum; WM, white matter.

Authors	Year	Functional Outcomes	Sample Size	Entry Route
Bleecker et al. [26]	2007	Cerebral WM changes	61	All
Cecil et al. [27]	2008	Gray matter volume	157	All
Brubaker et al. [28]	2009	Myelination and axonal integrity	91	All
Seo et al. [29]	2014	Functional abnormalities in the frontoparietal working memory network	65	All
Takeuchi et al. [30]	2021	Microstructural properties of gray matter areas, and brain activity	920	All
Migneron-Foisy et al. [32]	2022	Alterations of the CC	89	All

**Table 3:** List of the selected studies in the narrative review that explored the association of Hg and Cu and neural motor areas. Abbreviations: CC, corpus callosum; BG, basal gaglia.

Authors	Year	Functional Outcomes	Sample Size	Entry Route
Pujol et al. [31]	2016	Alterations in BG structure and function	263	Inhalation
Migneron-Foisy et al. [32]	2022	Alterations in the CC	89	Food

## 4. Discussion

Although metal exposure has significant public health implications, its neurobiological consequences remain insufficiently explored. In particular, the existing body of neuroimaging research is limited, with most studies focusing predominantly on the associations between metal exposure, cognitive decline, and an elevated risk of developing Parkinson’s disease. Nonetheless, our comprehensive critical review reveals compelling evidence that exposure to metals—particularly Mn—is associated with discernible alterations in cerebral regions responsible for motor control. Specifically, the literature indicates that both structural and functional modifications occur in key motor areas, including the primary motor cortex and BG [14,17,18,19], following metal exposure. Many studies employed R1 relaxometry because of its sensitivity to the paramagnetic effects of metal deposits, especially Mn, allowing for precise quantification of metal accumulation in the brain. This technique is particularly effective in detecting Mn buildup in regions predisposed to metal accumulation, such as the thalamus, putamen, pallidum, substantia nigra [23], and BG [22]. Furthermore, the study revealed statistically significant positive correlations between elevated R1 values in the cerebellum and frontal cortex and the Mn-CEI3M index, which is indicative of increased Mn accumulation. In particular, higher R1 values in the cerebellum were significantly associated with impaired motor performance, as measured by the UPDRS in welders. Given the cerebellum’s pivotal role in motor coordination and function, these findings underscore its vulnerability to Mn-induced neurotoxicity. Notably, there is a relative scarcity of studies addressing Mn deposition in the cerebellum and its direct toxic effects in humans. Previous studies have reported that Mn-related volumetric decreases in the cerebellum correlate with diminished performance in fine motor tasks and executive functions in full-time welders, highlighting the clinical implications of such exposures [14]. Among the analyzed articles, only one older review [25] addressed the neurotoxic effects of Mn exposure in workers. It concluded that Mn may accumulate in the brain after a critical exposure threshold, even at low levels, leading to microstructural changes. Synergy metrics may help detect early motor decline, and the role of other welding-related metals should also be considered. VBM has not been widely applied in studies of Mn neurotoxicity, despite some evidence of structural alterations in welders, particularly in the GP and cerebellum [14]. The limited use of VBM in this context may be attributed to methodological challenges inherent in imaging Mn-exposed populations. One major issue is the elevated T1W signal intensity caused by Mn accumulation, which can obscure anatomical boundaries and complicate the accurate segmentation of BG structures. This signal hyperintensity introduces potential confounds in volumetric analyses, thereby reducing the reliability and interpretability of morphometric findings in individuals with high Mn exposure. While occupational exposure to metals has been extensively studied, environmental exposure during early development remains underexplored. Emerging evidence indicates that adolescents and children exposed to elevated levels of metals, particularly Mn, exhibit altered iFC in key brain regions [16]. Specifically, increased iFC has been observed between the middle frontal gyrus and the medial prefrontal cortex, alongside decreased connectivity between the right putamen and the precentral and postcentral gyri. Given the putamen’s critical role in motor control, these disruptions are noteworthy. Previous studies have linked reduced iFC between the putamen and precentral gyrus to impaired motor function in children [33]. Neuroanatomical alterations in the BG subnuclei induced by Mn exposure are particularly significant in children exposed to Mn-contaminated drinking water [17]. Structural MRI studies have reported volumetric enlargements in several BG subregions, notably the left caudate nucleus, the entire putamen, and the GP. The concurrent involvement of these three interconnected regions suggests potential disruptions in the BG motor circuitry. Given that the brain undergoes substantial developmental changes from childhood through adolescence—characterized by dynamic processes of synaptogenesis, pruning, and myelination—the interaction between environmental neurotoxicants and normative neurodevelopment may result in distinct patterns of brain morphometry. These findings underscore the importance of considering the timing of Mn exposure, as the age at onset of neurotoxicity may critically influence the nature and extent of structural brain alterations. Collectively, these findings suggest that higher Mn exposure during early postnatal development may contribute to atypical connectivity patterns associated with deficits in both cognitive and motor domains.

The heightened vulnerability of motor-related brain regions to Mn neurotoxicity stems not merely from their role in motor regulation but from distinct neurobiological characteristics that make them particularly susceptible to toxic accumulation. Motor structures such as the BG, cerebellum, and corticospinal tracts exhibit exceptionally high metabolic activity, leading to increased oxidative stress and a greater demand for energy-dependent detoxification processes. Furthermore, these regions are rich in dopaminergic and GABAergic neurons, which are particularly sensitive to Mn dysregulation, as Mn influences neurotransmitter synthesis and synaptic function. Additionally, selective transport mechanisms—such as divalent metal transporters—facilitate Mn entry into neurons, resulting in preferential accumulation in motor-related areas. While alternative access routes, such as the olfactory epithelium, have been discussed in relation to toxicant exposure, their direct contribution to motor system impairment remains limited. Therefore, a more precise understanding of the interplay between vascular distribution, metal transport dynamics, and glial interactions is crucial for elucidating why motor regions—rather than associative or sensory areas—exhibit increased susceptibility to Mn toxicity. Although Mn has received considerable attention in neurotoxicological research, the neurobiological consequences of Pb exposure—particularly in relation to motor function—are increasingly recognized as a critical area of concern. Several neuroimaging studies have begun to elucidate the structural and functional brain alterations associated with both chronic and early-life Pb exposure, revealing a multifaceted impact on white [26,28,30] and gray matter integrity [27] (cerebellum, corona radiata, and CC), as well as on motor-related neural circuits [29]. Collectively, these studies provide converging evidence that Pb exposure—whether chronic, developmental, or low-level—can disrupt brain regions and pathways essential for motor function. These findings underscore the need for continued investigation into how Pb exposure affects brain function and development, particularly in vulnerable populations such as children. Emerging studies suggest that even low-level environmental exposure to metals such as Cu and Hg may have measurable impacts on brain structure and function, especially in children. MRI analyses for Cu- exposed subjects indicated reduced microstructural integrity in the caudate nucleus, a key component of the BG involved in motor control [31]. Furthermore, fMRI revealed diminished connectivity between the caudate and frontal cortex, suggesting that Cu exposure may impair frontostriatal communication—an essential pathway for motor planning and execution. Instead, prenatal and postnatal Hg exposure were linked to increased FA in posterior CC regions, especially the splenium [32]. This increase was mainly due to reduced RD, suggesting changes in myelination or axonal density. While higher FA often indicates better WM integrity, in this context, it may reflect atypical or compensatory development. Together, these studies broaden the scope of metal neurotoxicity research by demonstrating that metals beyond Mn and Pb—such as Cu and Hg—can also affect motor-related brain regions through both structural and functional pathways. They also emphasize the importance of early-life exposure windows, during which the brain is particularly susceptible to environmental insults. In addition to the lack of studies exploring the neurobiological consequences of single metals, especially related to the neuroimaging research focusing on motor control, there is a complete absence of studies exploring the combined effects of multiple metal exposures on the neural motor system. Individuals are rarely exposed to a single metal in a real- world setting; rather, they encounter complex mixtures of neurotoxicants like metals through air, water, food, and occupational sources. The motor network, comprising cortical and subcortical regions such as the primary motor cortex, basal ganglia, cerebellum, and frontal white matter, is highly complex, and its investigation benefits from the integration of diverse neuroimaging techniques. Relaxometry and T1W imaging reveal Mn accumulation in key motor regions, while VBM has identified gray matter volume reductions in the globus pallidus and cerebellum. DTI has shown decreased fractional anisotropy in the corpus callosum and frontal white matter, suggesting microstructural disruption of motor pathways. MRS adds a biochemical dimension, revealing altered levels of GABA and myo-inositol that are associated with motor performance. The heightened vulnerability of motor regions to metal toxicity is rooted in their high metabolic activity, dense dopaminergic and GABAergic innervation, and specialized metal transport mechanisms. These features promote metal accumulation and oxidative stress, contributing to neurodegeneration. Such mechanisms may explain why disorders like parkinsonism are more strongly linked to environmental toxins. Early-life exposure further amplifies risk by disrupting critical neurodevelopmental processes. Research on individual variability in motor system function helps explain why some people are more vulnerable to the neurotoxic effects of metal exposure. Moreover, individual differences in visuomotor learning and working memory capacity can offer important insights into how people respond differently to metal exposure [34]. Visuomotor learning involves the integration of visual information with motor actions skills that are often disrupted by neurotoxicants like manganese or lead. Similarly, working memory plays a key role in coordinating complex motor tasks and adapting to new motor demands. Metals may interact synergistically, additively, or antagonistically, potentially altering their individual effects [35] on motor-related brain regions. The lack of neuroimaging studies examining metal mixtures limits our understanding of how co-exposure influences WM integrity, BG structure, or frontostriatal and cerebellar circuits. Furthermore, without data on cumulative or interactive toxicological effects, risk assessments may underestimate the true burden of environmental metal exposure on neurodevelopment and motor function. Addressing this gap through multi-metal neuroimaging studies is critical for developing more accurate models of environmental neurotoxicity and informing public health interventions. Another important limitation observed across much of the existing literature is the small sample size employed in the majority of studies. Only three studies (15%) [27,30,31] included more than 100 participants, and just one study (5%) involved a large cohort of nearly 1000 subjects [30]. This raises significant concerns regarding the generalizability and statistical robustness of the reported findings. Small sample sizes limit the ability to detect neurotoxic effects, increase the risk of type I and type II errors, and reduce confidence in the replicability of the results. This highlights the need not only for more research in this area but also for studies designed with adequately powered samples to ensure more reliable and representative conclusions. The overrepresentation of Mn studies in the literature, compared to other neurotoxic metals, introduces a potential bias that may limit the generalizability of findings across different exposure contexts. While Mn has been extensively studied due to its known accumulation in motor-related brain regions, this focus may overshadow the neurotoxic effects of other metals such as lead, copper, or mercury, which also impact motor and cognitive functions. Additionally, technical limitations in neuroimaging methods, particularly VBM, must be acknowledged. Mn deposition can cause T1-weighted signal hyperintensities, which obscure anatomical boundaries and complicate the segmentation of subcortical structures like the BG. Given the diverse neurotoxic profiles of various metals, there is a pressing need for more comprehensive investigations utilizing advanced imaging modalities to elucidate a broader spectrum of neural alterations. Such research could uncover additional neuropathological features related to metal exposure, including changes in brain structure, functional connectivity, motor fMRI task, and neurochemical dynamics, thereby addressing a critical gap in our current understanding and informing preventative strategies and public health policies. Finally, a limitation noted in some of the reviewed studies is the lack of control for confounding variables. For example, in studies involving welders, factors such as age, socio-economic status, smoking status, and concurrent exposure to other metals or chemicals may confound the relationship between the exposure of interest and the observed neuroimaging outcomes. The inclusion of this information in future studies will improve the reliability of the results.

Future research should prioritize longitudinal study designs that can track neurodevelopmental and neurodegenerative changes over time. This will also help to establish causal links between metal exposure and brain changes: all the present studies present a cross- sectional design, which prevents us from proving causality and only allows us to assess associations. Additionally, the adoption of harmonized exposure metrics—including standardized biomarkers, cumulative exposure indices, and consistent environmental monitoring protocols—would enhance comparability across studies and improve meta-analytic power. Advanced neuroimaging techniques as Quantitative Susceptibility Mapping and Susceptibility-Weighted Imaging—iron sensitive—can provide more precise assessments of metal deposition, particularly in iron-rich regions like the BG and substantia nigra. Given the complexity of real-world exposures, future studies should also investigate the combined effects of multiple metals using mixed modeling approaches and machine learning techniques to identify synergistic or antagonistic interactions. This is especially important for understanding how co-exposures influence motor circuits, WM integrity, and frontostriatal–cerebellar connectivity.

The findings from neuroimaging studies have direct implications for intervention strategies. In occupational settings, enhanced workplace safety policies—such as improved ventilation, personal protective equipment, and regular biomonitoring—can help reduce exposure levels among high-risk groups like welders and smelters. In cases of elevated body burden, chelation therapy may be considered, although its efficacy and safety vary depending on the metal and individual health status. Therefore, chelation should be guided by clinical evaluation and supported by imaging biomarkers that reflect neurotoxic burden and treatment response. Special attention should be given to vulnerable populations, such as children and pregnant women, who are more susceptible to the neurodevelopmental effects of metal exposure. Finally, the development of early screening tools using neuroimaging biomarkers—such as elevated R1 values or altered functional connectivity—could facilitate the identification of individuals at risk before clinical symptoms emerge, enabling timely intervention and potentially preventing irreversible damage.

## 5. Conclusions

Although Mn and Pb have been the primary focus of metal neurotoxicity research, emerging evidence shows that other metals like Cu and Hg also impact brain regions involved in motor control. Neuroimaging studies reveal that exposure to these metals—especially during early development—can alter both brain structure and function, affecting areas such as the BG, cerebellum, and CC. Despite these findings, research remains limited, particularly regarding the combined effects of multiple metal exposures, which more accurately reflect real-world environmental conditions. Addressing this gap is essential for understanding the full scope of metal-induced neurodevelopmental risks.

## Data Availability

The data are publicly available.
